# Diagnostic data for neurological conditions in interRAI assessments in home care, nursing home and mental health care settings: a validity study

**DOI:** 10.1186/1472-6963-13-457

**Published:** 2013-11-01

**Authors:** Andrea D Foebel, John P Hirdes, George A Heckman, Marie-Jeanne Kergoat, Scott Patten, Ruth Ann Marrie

**Affiliations:** 1 School of Public Health & Health Systems, University of Waterloo, Waterloo, ON, Canada; 2 Institut universitaire de gériatrie de Montréal, Montreal, QC, Canada; 3 Department of Community Health Sciences, University of Calgary, Calgary, AB, Canada; 4 Departments of Medicine and Community Health Sciences, University of Manitoba, Winnipeg, MB, Canada

**Keywords:** Diagnostic validity, Nursing homes, Home care, Psychiatry, interRAI

## Abstract

**Background:**

The interRAI suite of assessment instruments can provide valuable information to support person-specific care planning across the continuum of care. Comprehensive clinical information is collected with these instruments, including disease diagnoses. In Canada, interRAI data holdings represent some of the largest repositories of clinical information in the country for persons with neurological conditions. This study examined the accuracy of the diagnostic information captured by interRAI instruments designed for use in the home care, long-term care and mental health care settings as compared with national administrative databases.

**Methods:**

The interRAI assessments were matched with an inpatient hospital record and emergency department (ED) visit record in the preceding 90 days. Diagnoses captured on the interRAI instruments were compared to those recorded in either administrative record for each individual. Diagnostic validity was examined through sensitivity, specificity and positive predictive value analysis for the following conditions: multiple sclerosis, epilepsy, Alzheimer’s disease and other dementias, Parkinson’s disease, traumatic brain injury, stroke, diabetes mellitus, heart failure and reactive airway disease.

**Results:**

In the three large study samples (home care: n = 128,448; long-term care: n = 26,644; mental health: n = 13,812), interRAI diagnoses demonstrated high specificity when compared to administrative records, for both neurological conditions (range 0.80 – 1.00) and comparative chronic diseases (range 0.83 – 1.00). Sensitivity and positive predictive values (PPV) were more varied by specific diagnosis, with sensitivities and PPV for neurological conditions ranging from 0.23 to 0.94 and 0.14 to 0.77, respectively. The interRAI assessments routinely captured more cases of the diagnoses of interest than the administrative records.

**Conclusions:**

The interRAI assessment collected accurate information about disease diagnoses when compared to administrative records within three months. Such information is likely relevant to day-to-day care in these three environments and can be used to inform care planning and resource allocation decisions.

## Background

Persons with chronic health problems, including neurological conditions, often receive health and social services across a broad continuum of care. Integration of health information from multiple service sectors is essential to support continuity of care. interRAI is an international, not-for-profit research network that was initially established by collaborating clinicians and researchers to improve quality of care and quality of life in nursing home settings [1], following the introduction of the first instrument in United States nursing homes [2,3]. After its initial implementation in the US, adoption of the Resident Assessment Instrument (RAI 2.0), and its successor, the interRAI Long Term Care Facility (LTCF) assessment, has occurred in numerous countries, including Canada [3-5].

The interRAI family of assessment instruments was designed to provide a common, integrated approach to standardized assessment of vulnerable populations with complex care needs, including those receiving home care, nursing home and mental health services [6-9]. The interRAI Home Care (RAI-HC) instrument was developed in the mid-1990s to establish a standardized assessment practice for individuals in community-based care settings [10]. It is now mandated for use in eight provinces/territories in Canada [11], and several other countries [1]. In psychiatric inpatient settings, the interRAI Mental Health (RAI-MH) instrument was designed for use in general adult psychiatry settings, including geriatric psychiatry [12,13]. Using a set of core items, each instrument collects comprehensive information about client demographic characteristics, functional status, clinical conditions, care needs, strengths and preferences allowing comparisons across sectors. Further, the instruments contain setting-specific items that allow tailoring of care plans to population needs in different care settings. An updated and expanded suite of interRAI instruments was developed beginning in 2000 to further streamline and integrate core items across all instruments, improve access to currently underserved populations and provide compatible assessment approaches for nursing homes, home care, community mental health and others [8,9]. These instruments assess individuals’ needs, strengths and preferences and they yield data that may be used for multiple applications by multiple stakeholders, including care planning [14], outcome measurement [15], quality improvement [16-20], and resource allocation [21-23].

The original interRAI assessment instruments and those in the new suite have demonstrated excellent reliability and validity across various care settings [7,8,12,24-30]. However, most of the psychometric research to date has dealt with the clinical items dealing with functional status, health symptoms, and psychosocial indicators. By comparison, little has been published about the performance of diagnostic items from these instruments. Each interRAI instrument contains a section to record disease diagnoses relevant to the individual’s status. Such diagnoses may be recorded using a “pick list” of items for common conditions, or by free text and ICD-10-CA codes for less common conditions [31-33]. Research on the validity of diagnostic items in the interRAI suite has focused primarily on long-term care settings, where diagnostic information was shown to be valid for a number of chronic and neurological conditions [34,35]. However, no information has been published to date on the validity of diagnoses recorded in these assessments in home care and mental health settings.

Recently, the Public Health Agency of Canada and the Neurological Health Charities of Canada funded the National Population Health Study of Neurological Conditions (NPHSC) to “provide a clear picture of the state of neurological conditions in Canada that will help governments and stakeholders plan programs and health services for Canadians living with these conditions and identify the scope for prevention” [36]. The data repositories based on interRAI assessment systems are of particular interest because they provide a large source of clinical data for persons with conditions like epilepsy, multiple sclerosis, Alzheimer’s disease and other dementias and Parkinson’s disease across the continuum of care.

This study addresses the level of agreement between interRAI assessments and the diagnostic information recorded in other datasets related to prior emergency department (ED) visits and hospital stays. This study sought to estimate the validity of selected neurological and chronic disease diagnoses recorded on the RAI 2.0, the RAI-HC and the RAI-MH through linkage with two national administrative databases in Canadian provinces (British Columbia, Manitoba, Newfoundland, Nova Scotia, Ontario, Saskatchewan) and territories (Yukon) submitting their data to the Canadian Institute for Health Information (CIHI). Evidence is provided for some of the key neurological conditions of interest in the NPHSC, but non-neurological diagnoses are also considered for comparative purposes.

## Methods

Data for this study were obtained as part of the *innovations in data, evidence and applications for Persons with Neurological Conditions* (ideas PNC) project. This national project made use of RAI-HC data collected from home care agencies in two provinces (Ontario and Nova Scotia) and one territory (Yukon); RAI 2.0 data collected from long-term care (LTC) facilities and complex continuing care (CCC) hospitals/units in six provinces (British Columbia, Manitoba, Newfoundland, Nova Scotia, Ontario, and Saskatchewan) and one territory (Yukon); and RAI-MH data collected from Ontario psychiatric hospitals/units. Using these databases, linkage with CIHI’s Discharge Abstract Database (DAD) and National Ambulatory Care Reporting System (NACRS) was done to examine diagnostic validity. The following neurological diagnoses were of interest: multiple sclerosis (MS), Alzheimer’s disease and other dementias (referred to collectively as dementias), epilepsy, Parkinson disease (PD), traumatic brain injury (TBI), and stroke (including transient ischemic attacks). To provide a comparative context for evaluating the performance of the neurological diagnosis items, other conditions examined included: diabetes mellitus, heart failure, and reactive airway diseases (including asthma, emphysema and chronic obstructive pulmonary disease [COPD]). This research study and the use of anonymized data were approved by the University of Waterloo’s Office of Research Ethics (ORE # 17045).

### Data and sample populations

The interRAI assessment data were obtained from three national repositories managed by CIHI (http://www.cihi.ca): the Home Care Reporting System (HCRS), the Continuing Care Reporting System (CCRS) and the Mental Health Reporting System (MHRS) [37-39]. These reporting systems collect information gathered using the RAI-HC, RAI 2.0 and RAI-MH instruments, respectively. Provinces contributed to the study samples in different proportions given different population sizes and different degrees of interRAI instrument implementation. Ontario is the country’s most populated province, the first to adopt interRAI instruments, and has achieved the fullest implementation in all sectors of interest. Therefore, assessments from Ontario represented most of the HC (88%), LTC/CCC (85%) and MH (93%) samples from which data for the current study were drawn. CIHI sets reporting standards and provides data quality checks for all submissions from participating provinces and territories. After submission, unique identifiers are created to de-identify individuals and allow for linkage with other administrative databases, and across interRAI assessments. Data were made available for this study based on an existing data-sharing agreement between CIHI, interRAI and the University of Waterloo.

**Figure 1:**
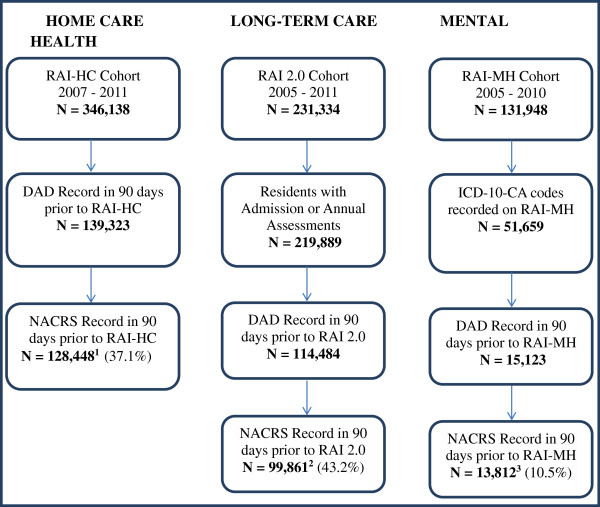
**Sample selection in home care, long-term care and mental health settings. **^1^Includes assessments from Ontario (99.9%) and Yukon Territory (0.1%). ^2^Includes assessments from British Columbia (0.1%), Ontario (99.8%) and Yukon Territory (0.1%). ^3^All assessments are from facilities in Ontario.

The CCRS data included 1,577,614 RAI 2.0 assessments collected between 2003 and 2011, representing 338,570 long term care residents. CCRS data are collected from both LTC and CCC hospitals/units, but this sample consisted mainly of LTC facility residents. The HCRS data holdings contained 864,955 assessments (from 502,257 clients) completed between 2001 and 2011. Lastly, the MHRS data included 470,586 assessments (representing 131,948 patients) from 70 psychiatric hospitals/units in Ontario between 2005 and 2010. Since instruments were mandated at different times, data from all three care settings were examined to determine a common period of interest during which consistent reporting was observed. CCRS data from 2005–2011, HCRS data from 2007–2011 and MHRS data from 2005–2010 were reported consistently and widespread implementation in all sectors of interest was considered well underway in those time periods. The samples were then limited to these periods for the respective datasets for the present analyses. From each of these datasets, persons with an interRAI assessment completed within 90 days after an ED visit or hospitalization were included (refer to Figure 1).

### Acute care data

The DAD has been shown to be valid across a number of conditions, with very high concordance reported in comparison with chart information [40-42]. Disease diagnoses from in-patient hospitalizations were extracted from the CIHI DAD. In this database, each record contains information about all diagnoses that the health record technologist obtained from individual hospital records. Most responsible diagnoses (primary diagnoses) are identified as those which were most responsible for the person’s acute hospital length of stay. In addition to this single primary diagnosis, each record may contain up to 24 other diagnoses (in some jurisdictions), recorded using the International Classification of Diseases version 10 Canadian Enhancement (ICD-10-CA) diagnostic codes [37].

### Emergency department data

The CIHI NACRS database was used to identify neurological diagnoses recorded during ED visits. Similar to the DAD, each record contains information about all diagnoses that health record technologists obtain from individual acute care charts. The diagnoses most responsible for the ED visit and up to nine other diagnoses were recorded for each client using ICD-10-CA diagnostic codes [43].

### Data collected from interRAI instruments

The interRAI instruments, including the RAI-HC, the RAI 2.0 and the RAI-MH, contain a core set of items to comprehensively describe client demographics and clinical characteristics to assist with care planning. Items specific to each type of care setting are also included in the instruments. For example, the items for substance use, excessive behaviours and harm to self and or others are included on the RAI-MH [33]. To capture disease diagnoses, all instruments contain items in two formats. First, pick lists of conditions that are relatively common in the particular care setting are available. For less common conditions, a free-text section is provided to record the name of the condition and the corresponding ICD-10-CA code. Trained assessors, often nurses or social workers, use all sources of information available, including interviews with the person and family members, consultation with other clinicians, and chart review to complete all sections of the interRAI instruments, including diagnosis. The assessor uses clinical judgment to reconcile inconsistent evidence from different sources. There are subtle differences in how disease diagnoses are captured between the RAI-HC, the RAI 2.0 and the RAI-MH, and these are discussed in the following sections.

### RAI-HC data

RAI-HC assessments are performed on long stay home care clients expected to receive services for 60 days or more, including those receiving maintenance and supportive care [11]. Reassessments are completed semi-annually in some provinces and annually in others. While most long-stay clients are assessed in the community, some are assessed in hospital to facilitate placement into LTC facilities. The RAI-HC instrument contains check-box items for disease diagnoses (section J) that are to be recorded for “Disease/infection that doctor has indicated is present and affects client’s status, requires treatment, or symptom management” [31]. Disease diagnoses should also be checked if the disease is monitored by a home care professional or led to a hospitalization in the 90 days prior to the assessment [31]. For this instrument, other current or more detailed diagnoses may be recorded using free-text and ICD-10-CA codes, but these codes were not available in the linked data set obtained from CIHI for this study. A diagnosis of epilepsy could not be obtained for the home care sample because it was not included in the diagnostic pick list in the RAI-HC. All other diagnoses were available Additional file 1: Table S1 describes in detail how this information was collected.

### RAI 2.0 data

The RAI 2.0 instrument is used to assess all individuals admitted to LTC or CCC facilities who are expected to stay for longer than 14 days. In section I1, disease diagnoses can be recorded using a check-box for 47 different diseases. Conditions are to be recorded if they “have a relationship to current activities of daily living (ADL) status, cognitive status, mood and behaviour status, medical treatments, nurse monitoring, or risk of death” [32], meaning that inactive diagnoses are not recorded. Section I3 allows for the inclusion of other current diagnoses by free-text and ICD-10-CA code. All neurological conditions of interest were available from the RAI 2.0 data, and both pick list items and ICD-10-CA codes were used to identify cases. Also, only admission and annual assessments were included because they include the most comprehensive pick lists for the conditions of interest (refer to Figure 1).

### RAI-MH data

The RAI-MH is currently mandated for use in Ontario, though residents from other provinces may access these services. Assessments are performed at admission and discharge and at three-month intervals for patients with extended stays. Medical diagnoses relevant to patients’ status are captured in section I11, but no neurological conditions are listed here. Instead, neurological conditions and other diagnoses relevant to patients’ status may be recorded using free-text and ICD-10-CA codes [33]. Additionally, section Q allows assessors to 'select up to three provisional DSM-IV diagnoses determined by the psychiatrist/attending physician and rank them in order of importance as factors contributing to this admission’ [33]. This section includes a single item to indicate the presence of delirium/dementia/amnestic and other cognitive disorders. All neurological conditions of interest were available from this data source, but assessments in which no ICD-10-CA codes were recorded were excluded from (n = 80,289; refer to Figure 1).

### Diagnostic coding

Diagnoses from the interRAI assessments were linked with ICD-10-CA codes based on instrument documentation and clinical review [31-33,43]. Identification of relevant ICD-10-CA codes for all the neurological conditions being examined by NPHSC grants was provided by a Canadian Chronic Disease Surveillance System Neurological Conditions Working Group led by the Public Health Agency of Canada and including clinical and health services researchers from across Canada. The transition from the previous ICD-9 classification codes to ICD-10-CA codes was implemented in Canada beginning in 2001, with all provinces except Quebec having adopted the newer codes by 2005 [44]. Thus, for the time periods examined here, only ICD-10-CA codes were used to identify cases. Additional file 1: Table S1 contains the complete lists of diagnostic categories of interest and the associated ICD-10-CA codes, and Additional file 1: Table S2 illustrates the corresponding interRAI disease diagnoses items.

### Statistical analysis

Using ICD-10-CA codes, data from each care setting were linked first with DAD and NACRS records, resulting in three data samples. For individuals with more than one matching interRAI and DAD record, the DAD record closest to the date of the interRAI assessment was chosen, leaving only a single record per individual. These individuals were then matched with NACRS records, again, if the interRAI assessment date fell within 90 days of an ED visit. If individuals had more than one matching ED visit to the interRAI assessment of interest, the visit closest to the time of the interRAI assessment was chosen. This left a single record for each individual with a DAD and NACRS record matched to a single interRAI assessment. The DAD and NACRS records were considered as the reference standards for this study, and diagnostic information from the interRAI instruments were compared to these records. Cases were defined from the DAD and NACRS record if either record identified the diagnoses of interest.

Demographic characteristics were compared using Chi-square tests for the categorical variables of interest, and mean ages of the three cohorts were compared using one-way ANOVA. Other analyses included assessment of the sensitivity, specificity and positive predictive values (PPV) of the diagnostic coding on the interRAI instruments, though the authors recognize that not all diagnoses captured in interRAI instruments are necessarily related to ED visits or hospitalizations. Kappa coefficients were also calculated as an alternate measure of agreement between interRAI data and administrative data sources. Sensitivity analyses measured the percentage of clients with a neurological diagnosis recorded in either the DAD or NACRS record whose same diagnosis was captured in the relevant interRAI assessment. Specificity analyses measured the percentage of clients or residents without a relevant diagnosis indicated on the DAD or NACRS who were noted as not have the relevant diagnosis on the linked interRAI record. PPV analysis measured the likelihood that an individual with an interRAI-coded diagnosis of a neurological condition had the same condition recorded on the DAD or NACRS record. All calculations were computed using 2 × 2 tables for each diagnosis which summarized the number of cases where an interRAI record did or did not record the neurological condition that was coded on the DAD or NACRS. For sensitivity, specificity and PPV estimates, 95% confidence intervals (CI) were calculated using a binomial distribution. All analyses were carried out using SAS version 9.2 (SAS Institute Inc., Cary, NC).

## Results

Table 1 shows the descriptive characteristics of the six samples studied. The patients in MH settings were the youngest (mean [SD] age 51.4 [17.9] years), while those in LTC/CCC settings (the RAI 2.0 samples) were the oldest (mean [SD] age 79.5 [11.7] years). One-way ANOVA analysis revealed the mean ages of the three cohorts to be significantly different (F value 11.99, p = 0.0029). Home care clients were slightly younger (mean [SD] age 77.4 [13.1] years) than persons in LTC/CCC. The HC and LTC/CCC samples were predominantly female, while the gender distribution was almost evenly distributed in the MH setting. More of the HC clients were married compared with persons in the other two settings. The three cohorts differed significantly with respect to age, gender and marital status according to results of the Chi-Square analyses.

**Table 1:** General characteristics of home care (N = 128,237), long-term care/complex continuing care (N = 99,861) and mental health (N = 13,795) populations

	**Home care**^**1 **^**(N = 128,448) n (%)**	**LTC/CCC**^**2 **^**(N = 99,861) n (%)**	**Mental health**^**3 **^**(N = 13,812) n (%)**	**Chi-square statistic (X**^**2**^**)**	**P value**
**Age**					
Less than 65 years	19,459 (15.2)	10,965 (11.0)	10,411 (75.4)		
65 – 74 years	20,123 (15.7)	14,324 (14.3)	1,603 (11.6)		
75 – 84 years	46,809 (36.4)	36,070 (36.1)	1,373 (9.9)	31,107.10	<.0001
Over 85 years	42,049 (32.7)	38,502 (38.6)	425 (3.1)		
**Gender**					
Female	78,210 (60.9)	61,461 (61.6)	7,422 (53.7)	342.52	<.0001
**Marital status**					
Married^4^	52,460 (40.8)	35,015 (35.1)	4,229 (30.6)	989.00	<.0001

^1^Assessments from Ontario (99.9%), Yukon (0.1%), 2007–2011; missing age for 8 individuals (<0.01%); missing marital status for 1 individual (<0.01%).

^2^Assessments from British Columbia (0.1%), Ontario (99.8%) and Yukon (0.1%), 2005–2011; missing marital status for 35 individuals (0.13%).

^3^Assessments from Ontario facilities, 2005–2010; missing marital status for 573 individuals (4.2%).

^4^Includes only individuals identified as 'married’ as this was consistent across instruments.

*Abbreviations*: *CCC* Complex continuing care hospitals/units, *LTC* Long-term care homes.

### Home care setting

The prevalence of the diagnoses coded on the RAI-HC and DAD or NACRS records, and the agreement of the RAI-HC records with DAD and NACRS records are shown in Table 2. For the neurological diagnoses with available data (epilepsy was not included in the RAI-HC pick list), dementias were the most prevalent condition. The RAI-HC captured more cases of neurological diagnoses than either administrative record. Using diagnoses recorded on the DAD or NACRS as a reference standard, two of four neurological diagnoses recorded on the RAI-HC had a sensitivity of at least 0.80, while another had a sensitivity greater than 0.70. Specificity for all neurological diagnoses of interest was high (>0.89), while PPVs were less consistent, ranging from 0.22 (TBI) to 0.77 (multiple sclerosis). Kappa coefficients were very high for MS (0.83), good for PD (0.68) and lower for the other neurological conditions examined.

**Table 2:** **Resident assessment instrument home care (RAI-HC) diagnoses**^1^**compared with hospital administrative record diagnoses, Ontario and Yukon, 2007–2011 (n = 128,448) - prevalence, sensitivity, specificity, positive predictive value and kappa**

	**RAI-HC cases % (n)**	**DAD or NACRS cases % (n)**	**Sensitivity (95% CI)**	**Specificity (95% CI)**	**PPV (95% CI)**	**Kappa (95% CI)**
Diabetes mellitus	27.5 (35,360)	27.9 (35,841)	.90 (.89, .90)	.96 (.96, .97)	.91 (.90, .91)	.86 (.86, .87)
Reactive airway disease^2^	20.9 (26,832)	12.3 (15,747)	.76 (.75, .76)	.87 (.87, .87)	.44 (.44, .45)	.48 (.47, .49)
Stroke	20.8 (26,666)	8.2 (10,579)	.76 (.76, .77)	.84 (.84, .84)	.30 (.30, .31)	.36 (.35, .37)
Alzheimer’s disease and other dementia	19.8 (25,464)	14.0 (17,996)	.76 (.75, .76)	.89 (.89, .89)	.53 (.53, .54)	.55 (.55, .56)
Heart failure	17.0 (21,883)	14.0 (17,922)	.61 (.60, .61)	.90 (.90, .90)	.50 (.49, .50)	.46 (.46, .47)
Parkinson disease	3.8 (4,847)	2.7 (3,451)	.83 (.81, .84)	.98 (.98, .99)	.59 (.58, .60)	.68 (.67, .69)
Traumatic brain injury	1.3 (1,716)	1.3 (1,665)	.23 (.21, .25)	.99 (.99, .99)	.22 (.20, .24)	.22 (.20, .24)
Multiple sclerosis	0.8 (1,071)	0.7 (923)	.90 (.87, .91)	1.00 -	.77 (.74, .80)	.83 (.81, .85)

^1^Refer to Additional file 1: Table S2 for items used for disease diagnoses on the RAI-HC.

^2^Includes: Asthma, Chronic Obstructive Pulmonary Disease (COPD), and Emphysema.

*Abbreviations*: *CI* Confidence interval, *DAD* Discharge abstract database, *K* Kappa statistic, *NACRS* National ambulatory care reporting system, *PPV* Positive predictive value, *RAI-HC* Resident assessment instrument – home care.

### Complex continuing care/long-term care setting

Table 3 shows the prevalence of recorded diagnoses and the agreement of the RAI 2.0 with DAD or NACRS records. Dementias were the most prevalent neurological condition. For all conditions except for TBI, the RAI 2.0 captured more cases than did administrative data. Sensitivities of the neurological diagnoses were generally high, ranging from 0.72 for epilepsy to 0.94 for MS, with the exception of TBI which had a sensitivity of 0.26. Specificities were generally high for all neurological conditions when the RAI 2.0 was compared with either administrative database, with four of five conditions having specificities of at least 0.96. PPVs were again, more variable, ranging from 0.14 for epilepsy to 0.77 for MS. Kappa coefficients were very high for MS (0.84), and good for PD (0.65), while agreement for dementias, TBI and epilepsy was lower (0.51, 0.28, 0.22, respectively).

**Table 3:** **Resident assessment instrument 2.0 (RAI 2.0) diagnoses**^**1 **^**compared with administrative record diagnoses, British Columbia, Ontario and Yukon, 2005–2011 (N = 99,861) - prevalence, sensitivity, specificity, positive predictive value and kappa**

	**RAI 2.0 cases % (n)**	**DAD or NACRS cases % (n)**	**Sensitivity (95% CI)**	**Specificity (95% CI)**	**PPV (95% CI)**	**Kappa (95% CI)**
Diabetes mellitus	27.4 (27,312)	25.6 (25,584)	.91 (.91, .91)	.95 (.94, .95)	.85 (.85, .86)	.84 (.83, .84)
Reactive airway disease^2^	20.0 (20,007)	10.7 (10,649)	.79 (.79, .80)	.87 (.87, .87)	.42 (.32, .43)	.48 (.47, .79)
Stroke	23.4 (23,401)	10.3 (10,331)	.81 (.80, .81)	.83 (.83, .83)	.36 (.35, .36)	.41 (.40, .42)
Alzheimer’s disease and other dementia	32.6 (32,591)	20.2 (20,182)	.83 (.82, .83)	.80 (.80, .80)	.51 (.51, .52)	.51 (.51, .52)
Heart failure	15.4 (15,418)	12.8 (12,789)	.61 (.61, .62)	.91 (.91, .92)	.51 (.50, .51)	.48 (.48, .49)
Parkinson disease	4.9 (4,901)	3.2 (3,163)	.85 (.84, .86)	.98 (.98, .98)	.55 (.53, .56)	.65 (.64, .67)
Traumatic brain injury	1.1 (1,066)	1.3 (1,343)	.26 (.23, .28)	.99 (.99, .99)	.32 (.29, .35)	.28 (.25, .30)
Multiple sclerosis	1.0 (990)	0.8 (802)	.94 (.93, .96)	.99 (.99, .99)	.77 (.74, .79)	.84 (.83, .86)
Epilepsy	4.7 (4,681)	0.9 (910)	.72 (.69, .75)	.96 (.96, .96)	.14 (.13, .15)	.22 (.21, .24)

^1^Refer to Additional file 1: Table S2 for items used for disease diagnoses on the RAI 2.0.

^2^Includes: Asthma, Chronic Obstructive Pulmonary Disease (COPD), and Emphysema.

*Abbreviations*: *CI* Confidence interval, *DAD* Discharge abstract database, *K* Kappa statistic, *NACRS* National ambulatory care reporting system, *PPV* Positive predictive value, *RAI 2.0* Resident assessment instrument version 2.0.

### Mental health setting

Table 4 shows the prevalence of conditions coded on the RAI-MH, DAD or NACRS records and the agreements of the RAI-MH with the administrative databases. Generally, prevalence of neurological diagnoses was lower compared with other care settings examined, but dementias were the most prevalent within this sector. Compared to administrative records, sensitivity of RAI-MH diagnoses were good for dementias (0.84) and MS (0.77), with lower values reported for the other neurological conditions. Specificities were very high (>0.92) for all neurological conditions, while PPVs ranged from 0.27 (epilepsy) to 0.55 (both PD and TBI). For the neurological conditions examined, Kappa coefficients indicated good agreement for MS (0.61), and moderate to fair agreement for other conditions.

**Table 4:** **Resident assessment instrument mental health (RAI-MH) diagnoses**^**1 **^**compared with administrative record diagnoses, Ontario, 2005–2010 (N = 13,812) - prevalence, sensitivity, specificity, positive predictive value and kappa**

	**RAI-MH cases % (n)**	**DAD or NACRS cases % (n)**	**Sensitivity (95% CI)**	**Specificity (95% CI)**	**PPV (95% CI)**	**Kappa (95% CI)**
Diabetes mellitus	16.0 (2,207)	15.7 (2,164)	.78 (.76, .80)	.96 (.95, .96)	.77 (.75, .79)	.73 (.72, .75)
Reactive airway disease^2^	9.9 (1,365)	4.4 (604)	.61 (.57, .65)	.93 (.92, .93)	.27 (.25, .30)	.34 (.31, .36)
Stroke	1.2 (170)	1.3 (178)	.20 (.16, .27)	.99 (.99, .99)	.21 (.15, .28)	.20 (.14, .25)
Alzheimer’s disease and other dementia	14.2 (1,959)	7.8 (1,083)	.84 (.82, .86)	.92 (.91, .92)	.46 (.44, .49)	.55 (.53, .57)
Heart failure	1.6 (226)	1.6 (224)	.42 (.35, .49)	.99 (.99, .99)	.42 (.35, .48)	.41 (.35, .47)
Parkinson disease	1.6 (218)	1.6 (223)	.54 (.47, .60)	.99 (.99, .99)	.55 (.48, .62)	.54 (.48, .59)
Traumatic brain injury	0.4 (62)	1.0 (132)	.26 (.19, .34)	1.00 -	.55 (.42, .68)	.35 (.26, .44)
Multiple sclerosis	0.6 (76)	0.4 (51)	.77 (.63, .87)	1.00 -	.51 (.40, .63)	.61 (.51, .71)
Epilepsy	2.9 (406)	2.0 (273)	.41 (.35, .47)	.98 (.98, .98)	.27 (.23, .32)	.31 (.26, .36)

^1^Refer to Additional file 1: Table S2 for items used for disease diagnoses on the RAI-MH.

^2^Includes: Asthma, Chronic Obstructive Pulmonary Disease (COPD), and Emphysema.

*Abbreviations*: *CI* Confidence interval, *DAD* Discharge abstract database, *K* Kappa statistic, *NACRS* National ambulatory care reporting system, *PPV* Positive predictive value, *RAI-MH* Resident assessment instrument – mental health.

## Discussion

This is the first study to examine the validity of diagnostic information collected in the home care and mental health settings using the RAI-HC and RAI-MH instruments when compared with both hospitalization and ED records. Further, this work is unique in its examination of the validity of a number of neurological conditions as well as comparison chronic diseases on the RAI 2.0 compared to both hospitalization and ED records.

In the home care and LTC/CCC settings, the most prevalent conditions were those associated with aging populations, such as dementia and stroke. Among the younger residents in the mental health setting examined, there were fewer cases of all conditions, and aging-related diagnoses such as dementias and stroke were less common. We found that when compared with inpatient and/or ED administrative records available in the 90 days prior to the index RAI assessment, the three interRAI tools have good sensitivity for some neurological diagnoses (particularly dementias and MS) and other chronic diseases (particularly diabetes mellitus). The high specificities shown by the RAI-HC, RAI 2.0 and RAI-MH instruments for all diagnoses examined provide strong evidence of the ability of the instruments to identify persons without the condition. The PPV values pertain to the issue of true positives in the assessments. The PPVs were generally very good for conditions like diabetes mellitus and MS, but weaker for traumatic brain injury, stroke, and epilepsy. Overall, the results reported here provide positive support for the accuracy of diagnostic information in interRAI assessments completed in standard practice.

In all three settings, interRAI instruments generally captured more cases than the administrative records. Diagnoses examined that would presumably be considered relevant for day-to-day care in HC, LTC/CCC and MH settings may not necessarily be responsible for, and subsequently recorded during, an ED visit or hospitalization. As such, they would be less likely to be observed in the DAD and NACRS records. Measuring comorbidities using administrative databases has been shown to be an area for improvement [45], and other work suggests that comorbidities are under-reported in administrative databases, including the DAD [46]. Thus, conditions not directly related to length of stay or ED visit may not be recorded even if they are present. In that sense, the DAD and NACRS may not represent true gold standards for detection of all of the conditions considered here. The poorer kappa and PPV values noted for some conditions may reflect this problem of under-reporting in acute care data sets.

The high sensitivities and specificities observed for some of the conditions strengthen the case for the accuracy of diagnostic information captured on interRAI assessments. In the HC and LTC/CCC settings, the sensitivities and specificities for all conditions except for TBI were above 0.61. These results compare well to the sensitivities found by Wodchis and colleagues using RAI 2.0 data [35]. The current study found similar specificities for stroke (0.83 vs. 0.84) and higher specificities for heart failure (0.91 vs. 0.80), PD (0.98 vs. 0.87), diabetes mellitus (0.95 vs. 0.88), reactive airway diseases (0.87 vs. 0.71), MS (0.99 vs. 0.20) and dementias (0.80 vs. 0.61 [Alzheimer’s] and 0.54 [non-Alzheimer’s dementia]) compared to this earlier work [35]. One explanation for the improvement in agreement is that the current study included diagnoses from both DAD and NACRS records, while earlier work focused solely on DAD data. Additionally, the current study included only a 3-month look back window, while Wodchis and colleagues examined a 6-month period.

Notably lower were the sensitivities and specificities for TBI. However, earlier work also found that sensitivity was low for this condition [35]. If this diagnosis was over-recorded on the DAD or NACRS record or under-reported on the interRAI instruments, this sensitivity would be lower than that for other conditions. However, there is good evidence of the validity of diagnostic coding on DAD records [40-42], and the interRAI instruments have demonstrated excellent inter-rater reliability [8,10,13,26]. Thus, coding errors of this nature may be minimal. One possibility is that, for this particular diagnosis, the check-box items on the interRAI instruments may be considered vague, making assessors less likely to record the diagnosis (see Additional file 1: Table S2 for interRAI diagnostic items that were used). There were fewer cases of TBI in the mental health setting and it is possible that it was under-recorded as no pick list items for neurological conditions existed in this data set.

The lower sensitivities observed in the mental health setting overall may partially reflect the fact that the concern of psychiatric care is psychiatric conditions and other somatic conditions would be less likely to be recorded during routine assessment with the RAI-MH. A second potential explanation is more methodological in nature - the RAI-MH contains only limited diagnoses in a pick list with check-box items. As stated, no check box items were available for neurological conditions, and ICD-10-CA codes were used for all neurological conditions examined in this study, as well as the more general item to identify dementia. Though trained in data abstraction and ICD-10-CA codes, it is possible that assessors record conditions using ICD-10-CA less frequently than by check-box items. In an attempt to limit this potential under-reporting of cases, only individuals with any ICD-10-CA code recorded on the RAI-MH were included in the samples.

Compared to the high sensitivities and specificities observed, PPVs were generally lower. The PPVs are influenced by prevalence of the condition in the respective sectors, but the variation may also be due in part to the tendency for hospital based administrative records to under-detect many of these conditions. A previous study by Gambassi and colleagues reported the PPVs of RAI 2.0 diagnostic codes compared with hospital discharge claims in the United States [34]. The reported PPVs for conditions examined in this earlier study were generally higher for some diseases, including PD (0.86 vs. 0.55) and dementias (0.68 [Alzheimer’s] vs. 0.51); TBI, MS and epilepsy were not included in the earlier work [34]. These discrepancies could reflect differences in recording of diagnoses in hospital records between the two countries or the longer period of time used to identify cases in the earlier study. However, the overall low PPVs in this study are likely due in large part to the nature of DAD and NACRS coding. Conditions most related to length of stay and resource use are likely to be preferentially recorded in these databases. Many of the conditions examined are chronic conditions that may not be directly responsible for a hospital or ED visit. Thus, such conditions in particular may be less likely to appear on administrative records, driving PPV estimates down. This would point to the interRAI instruments as a potentially more accurate reflection of comorbidity than DAD or NACRS records.

The current study has several limitations that should be noted. First, the data were collected from provinces and territories across Canada, and while there are standard assessment protocols for recording information in administrative data and interRAI assessments, there may be some regional variation in how diagnoses are reported. It is also worth noting that, while information was collected in different Canadian provinces and territories, Ontario represented both the largest sample within this study and the province with most uptake of interRAI instruments. Results may not be as generalizable in other parts of the country, particularly for the mental health settings, where facilities were all Ontario-based. Second, issues related to differences between diagnostic items for the conditions of interest between the RAI-HC, the RAI-MH and the RAI 2.0 may mean that assessors are more confident with some diagnoses than others. Where ICD-10-CA codes were used as the only source of disease identification (particularly in RAI-MH assessments), we cannot determine whether assessors were less likely to complete these items. For the RAI-HC data, it was not possible to capture epilepsy cases, as the HCRS data cut did not include the ICD-10-CA codes and there is no check-box item for epilepsy on the assessment itself. The provisional diagnosis of a cognitive disorder (item Q1b in the RAI-MH) was used for a proxy for dementia for RAI-MH data, but it does not deal exclusively with dementia, introducing the possibility that some of the identified cases were actually delirium or other cognitive issues. This would reduce the level of agreement between the RAI-MH and administrative data, subsequently reducing all three measures of diagnostic accuracy for that diagnosis in the RAI-MH. Third, this study examined a three month look back period prior to interRAI assessment. This short time frame was chosen specifically to gain insight about the accuracy of diagnostic information following an acute care encounter, but means that results will reflect the methodology chosen. Allowing for a longer look back period could potentially have allowed more cases to be identified from administrative records. Fourth, the current study did not examine whether all recorded conditions were active. The ability to compare diagnoses captured on the interRAI instruments with outpatient data (e.g., physician billing data), which would include more comprehensive patient profiles than administrative data, would be helpful in understanding further the accuracy of diagnostic information on interRAI assessments. Finally, while the current study indicates that there is good agreement between administrative data and interRAI diagnostic data, the interRAI items do not provide any information about disease severity, subtype, duration or signs and symptoms.

This study also had several important strengths. First, large sample sizes were obtained from all three care settings examined. While not necessarily representative of all residents in each setting, this sample size makes this validation study one of the largest to date, and includes vulnerable individuals from across the care continuum. This study was thus able to build on previous work by examining data in these care settings in comparison with two large, national administrative databases. Second, the data holdings examined in this study yielded some of the largest samples to date for neurological conditions in Canada, particularly the province of Ontario. Third, by identifying subtle differences in diagnostic items across interRAI assessments, this study has identified opportunities for improvement of current assessments, and areas in which training for assessors can be targeted to improve data collection. Additionally, by building on previous validation work, this study has begun to address the changing quality of data from interRAI instruments that have been achieved as tools are refined and redeveloped. There is good agreement with the prevalence of some conditions, such as heart failure, with other data from home care and long-term care settings in Canada [47] and internationally [48]. Finally, this study has provided evidence regarding potential problems with using administrative data as the standard to which to compare diagnostic information and included measures of agreement using Kappa coefficients. Without utilizing a long look back period, accurate reflections of current comorbidities may not be achievable using these traditional sources.

## Conclusion

In conclusion, these results indicate that the diagnostic data available on interRAI instruments for a number of neurological and chronic conditions show a high degree of accuracy and can be used with confidence for health research. Future work to link interRAI data to other outpatient and clinical data would further help to strengthen the case for its validity across a range of conditions. Utilizing interRAI data could help with further exploration of problems and outcomes in currently underserviced populations such as persons with neurological conditions and the frail elderly.

## Abbreviations

ADL: Activities of daily living; CCC: Complex continuing care; CCRS: Continuing care reporting system; CI: Confidence interval; CIHI: Canadian Institute for health information; COPD: Chronic obstructive lung disease; DAD: Discharge abstract database; ED: Emergency department; HC: Home care; HCRS: Home care reporting system; ICD-10-CA: International classification of diseases version 10 Canada; ideas PNC: Innovations in data, evidence and applications for persons with neurological conditions; LTC: Long-term care; MH: Mental health; MS: Multiple sclerosis; NACRS: National ambulatory care reporting system; NPHSC: National Population Health Study of Neurological Conditions; OMHRS: Ontario mental health reporting system; PD: Parkinson’s disease; PPV: Positive predictive value; RAI-HC: Resident assessment instrument – home care; RAI 2.0: Resident assessment instrument – 2.0; RAI-MH: Resident assessment instrument – mental health; TBI: Traumatic brain injury.

## Competing interests

The authors declare that they have no competing interests.

## Authors’ contributions

ADF and JPH jointly drafted the first version of the manuscript. JPH, GAH, MJK, SP, and RAM provided oversight to the research proposal and data acquisition. All authors contributed towards dataset preparation and analysis for this manuscript. All co-authors contributed toward conceptualization and revision of the manuscript, and have approved the final version.

## Pre-publication history

The pre-publication history for this paper can be accessed here:

http://www.biomedcentral.com/1472-6963/13/457/prepub

## Supplementary Material

Additional file 1: Table S1 Summary of ICD-10-CA Codes used for Conditions of Interest. **Table S2.** Disease Diagnoses as Captured using the RAI-HC, RAI 2.0 and RAI-MH. Click here for file
