# Supplementary material for: Diagnostic data for neurological conditions in interRAI assessments in home care, nursing home and mental health care settings: a validity study
Source: BMC Health Serv Res. 2013 Nov 1;13:457. doi: 10.1186/1472-6963-13-457 (PMC3893477; doi:10.1186/1472-6963-13-457)
Supplement: Additional file 1: Table S1 — Summary of ICD-10-CA Codes used for Conditions of Interest. Table S2. Disease Diagnoses as Captured using the RAI-HC, RAI 2.0 and RAI-MH. [file 1472-6963-13-457-S1.docx]

**ADDITIONAL FILES**

**Table 1:** **Summary of ICD-10-CA Codes used for Conditions of Interest**

| Conditions of Interest | ICD-10-CA Code(s) |
| --- | --- |
| Alzheimer’s disease &  other dementias | F00; F01; F02; F03; G30 |
| Diabetes Mellitus | E10; E11; E13; E14 |
| Epilepsy | G40 |
| Heart Failure | I50 |
| Multiple Sclerosis | G35 |
| Parkinsonism | F02.3; G20; G21; G22 |
| Reactive Airway Diseases^1^ | J43; J44; J45 |
| Stroke^2^ | G45.0; G45.1; G45.2; G45.3; G45.8; G45.9;  H34.1; I60; I61;  I63.0; I63.1; I63.2; I63.3; I63.4; I63.5; I63.7; I63.8; I63.9; I64 |
| Traumatic Brain Injuries | F07.2;  S02.0; S02.1; S02.3; S02.7; S02.8; S02.9;  S06; S07; S09.8;  T06.0; T90.5 |

^1^Includes a diagnosis of asthma, chronic obstructive pulmonary disease (COPD) and emphysema.

^2^Includes transient ischemic attacks (TIA)

**Abbreviations:** ICD-10-CA = International Classification of Diseases version 10 Canada.

**Table 2: Disease Diagnoses as Captured using the RAI-HC, RAI 2.0 and RAI-MH Instruments**

|  | RAI-HC^1^  2007-2011  N = 128,448 | RAI 2.0^2^  2005-2011  N = 99,861 | RAI-MH^3^  2005-2010  N = 13,812 |
| --- | --- | --- | --- |
| Alzheimer’s disease  & other dementias | J1g: Alzheimer’s  J1h: Dementia other than Alzheimer’s disease | I1r: Alzheimer’s disease  I1v: Dementia other than Alzheimer’s disease | Q1b: Delirium, dementia, amnestic and other cognitive disorders |
| Diabetes Mellitus | J1y: Diabetes | I1a: Diabetes Mellitus | I11a: Diabetes type 1  I11b: Diabetes type 2 |
| Epilepsy | N/A | I1cc: Seizure Disorder |  |
| Heart Failure | J1b: Congestive heart failure | I1f: Congestive heart failure | I11d: Congestive heart failure |
| Multiple Sclerosis | J1k: Multiple sclerosis | I1y: Multiple sclerosis |  |
| Parkinson’s disease | J1l: Parkinsonism | I1aa: Parkinson’s disease |  |
| Reactive Airway Diseases^4^ | J1z: Emphysema, COPD, asthma | I1jj: Asthma  I1kk: Emphysema, COPD | I11c: Chronic obstructive pulmonary disease |
| Stroke | J1a: Cerebrovascular accident (stroke) | I1u: Cerebrovascular accident (stroke)  I1dd: Transient ischemic attack (TIA) |  |
| Traumatic Brain Injuries | J1i: Head trauma | I1ee: Traumatic brain injury |  |

^1^ Assessments from Ontario, Yukon; all diagnoses except epilepsy determined using pick list items.

^2^ Assessments from British Columbia, Ontario, Yukon; all diagnoses determined using both pick list

items and ICD-10-CA codes.

^3^ Assessments from Ontario facilities; all diagnose determined using pick list items (where indicated)

and ICD-10-CA codes.
